# Exploring the impact of Chuangguandong Movement on individualism in China based on Sina Weibo information

**DOI:** 10.3389/fpsyg.2022.1046581

**Published:** 2023-01-04

**Authors:** Yan Wang, Xiaopeng Ren

**Affiliations:** ^1^Institute of Psychology, Chinese Academy of Sciences, Beijing, China; ^2^Department of Psychology, University of Chinese Academy of Sciences, Beijing, China

**Keywords:** individualism, voluntary frontier settlement hypothesis, Chuangguandong Movement, social media, Sina Weibo

## Abstract

The voluntary frontier settlement hypothesis holds that frontier movements can promote the formation of individualism in the frontier area. The Chuangguandong Movement is one of China’s voluntary frontier movements that potentially had a positive impact on the formation of high individualism in the northeastern provinces. Previous studies used independent/interdependent measures of self-construal scale, symbolic self-inflation, nepotism tasks, and percentage of most common names, to examine the differences in the independence between Heilongjiang and Shandong residents, which may be related to the Chuangguandong Movement. However, these studies were limited by certain factors such as sample size and objectivity of materials acquisition. In this study, we obtained Sina Weibo big data for period 2010–2020 to overcome the limitation of previous work. Using text feature extraction and keyword word frequency calculation methods based on the individualism/collectivism dictionary, we found that the level of individualism in Northeast China was higher than that in Shandong Province, which was consistent with previous research. Through the discussion of the four representative theoretical frameworks of individualism, the voluntary frontier settlement theory was considered as a potential explanation for the high degree of individualism in Northeast China.

## 1. Introduction

Individualism and collectivism are two important dimensions that measure the differences in values, which are usually used to indicate differences in tendencies toward social interactions. Individualism emphasizes the importance of “I,” focusing on one’s own uniqueness and autonomy, and caring more about oneself and immediate family. In contrast, collectivism emphasizes the importance of “we” and focuses on support and identity within the organization, caring more about the interests and cooperation of the collective. These are two separate dimensions that are not mutually exclusive, which has been proven not only by cross-cultural studies but also within the same cultures or within the same country (Oyserman et al., 2002; Su and Ren, 2014).

Differences in individualism/collectivism have also been found between various regions within China. Starting from the influencing factors of individualism/collectivism, the following theoretical frameworks are commonly used for the research and interpretation of differences within the same culture: (1) Modernization theory argues that the more modernized the region is, the higher the level of individualism. Conversely, the less modernized the region is, the higher the level of collectivism (Inglehart and Baker, 2000). The theory focuses on the impact of economic development on culture. Additionally, culture becomes more individualistic with economic growth and over time (Hamamura, 2012). (2) Climate-economic theory argues that the interaction between climate and economy may impact levels of individualism/collectivism. Van de Vliert (2011) found a correlation between climate and economic resource demand in climate-economic theory. When the climate environment is harsh and economic resources are insufficient, the collective ties between people are strengthened as this may promote survival. When the climate is comfortable, people have less need for the climate environment and are more inclined to form more independent relations. Van de Vliert et al. (2013) provided further support for this theory shortly afterward by studying the relationship between individualism/collectivism fractions, monetary resources (*per capita* household income), and climate demand. Their results showed that collectivism was strongest in low-income provinces with harsh climate environments (such as Heilongjiang), and collectivism was weakest in high-income provinces with suitable climate environments (such as Guangdong). (3) Rice theory argues that different modes of subsistence such as agriculture, forestry, animal husbandry and fishery have a certain influence on individualism/collectivism (Uskul et al., 2008). Talhelm et al. (2014) studied in rice-growing and wheat-growing regions and examined cultural thought, implicit individualism, and loyalty/nepotism, finding that rice-growing regional cultures were more collectivistic, while wheat-growing regional cultures were more individualistic. The present study further explored whether the above theories could explain the manifestations of high individualism in Northeast China.

Frederick Jackson Turner (1920) proposed the voluntary frontier settlement hypothesis, and Kitayama et al., (2006), Park (2007) further enriched the theory by positing that it involved three distinct psychological processes involved. First, frontier self-selection refers to settlers’ psychological motivation. People are driven by the desire for wealth and freedom, and they often possess a high degree of autonomy, independent psychological characteristics, and willingness to accept challenges. Second, the poor living conditions on the frontier reinforce the character of independence in the settlement process. The frontier life is often too harsh, full of uncertainty, and requires high level of self-reliance to promote survival. Simultaneously, there is no perfect system to protect people’s rights and interests, so people have to rely on themselves to solve various problems they face. In such environment, individual initiative is encouraged and strengthened. Third, a shared behavioral and spiritual culture gradually forms after settlement by gathering of likeminded people. This culture originates from settlers’ daily life, education, communication, and other characteristics, and it passes from generation to generation. Therefore, Kitayama et al. (2006) argued that the history of the United States is largely a history of the expansion of the Great West, which was mentioned as the birthplace of American history and had played a crucial role in shaping the American territory and the formation of the national character. It is this pioneering spirit that has nurtured and shaped America’s remarkably individualist culture. To test this hypothesis, Kitayama et al. (2009), Varnum and Kitayama (2011) compared the individualism in the United States to that of other countries. First, they compared North Americans (US), Western Europeans (British and German), and Asians by examining focused (vs. holistic) attention, the association of experienced emotions with independence (vs. interdependence), the association of happiness with personal achievement (vs. communal harmony), and an inflated symbolic self. Ultimately, they found that North Americans showed stronger individualistic tendencies than Western Europeans on all tests of implicit psychological disposition except for personality bias in attribution. Bazzi et al. (2020) also provided evidence about the roots of frontier culture by tracing it from 1790 to 1890, identifying a causal link between selective migration and frontier individualism. These findings further confirmed that frontier regions can create and strengthen individualism.

Does the voluntary frontier hypothesis also apply in collectivist China? Some researchers measured voluntary frontier settlement through the ongoing voluntary frontier region Shenzhen and the control region Foshan based on student groups using a one-sided measure intergroup design with a frame-line task and a sociogram task respectively, finding that Shenzhen had higher independent performance than the control region Foshan (Chen et al., 2016). The studies confirmed that the voluntary frontier settlement hypothesis is valid in China and could explain the impact on individualism.

**Figure 1:**
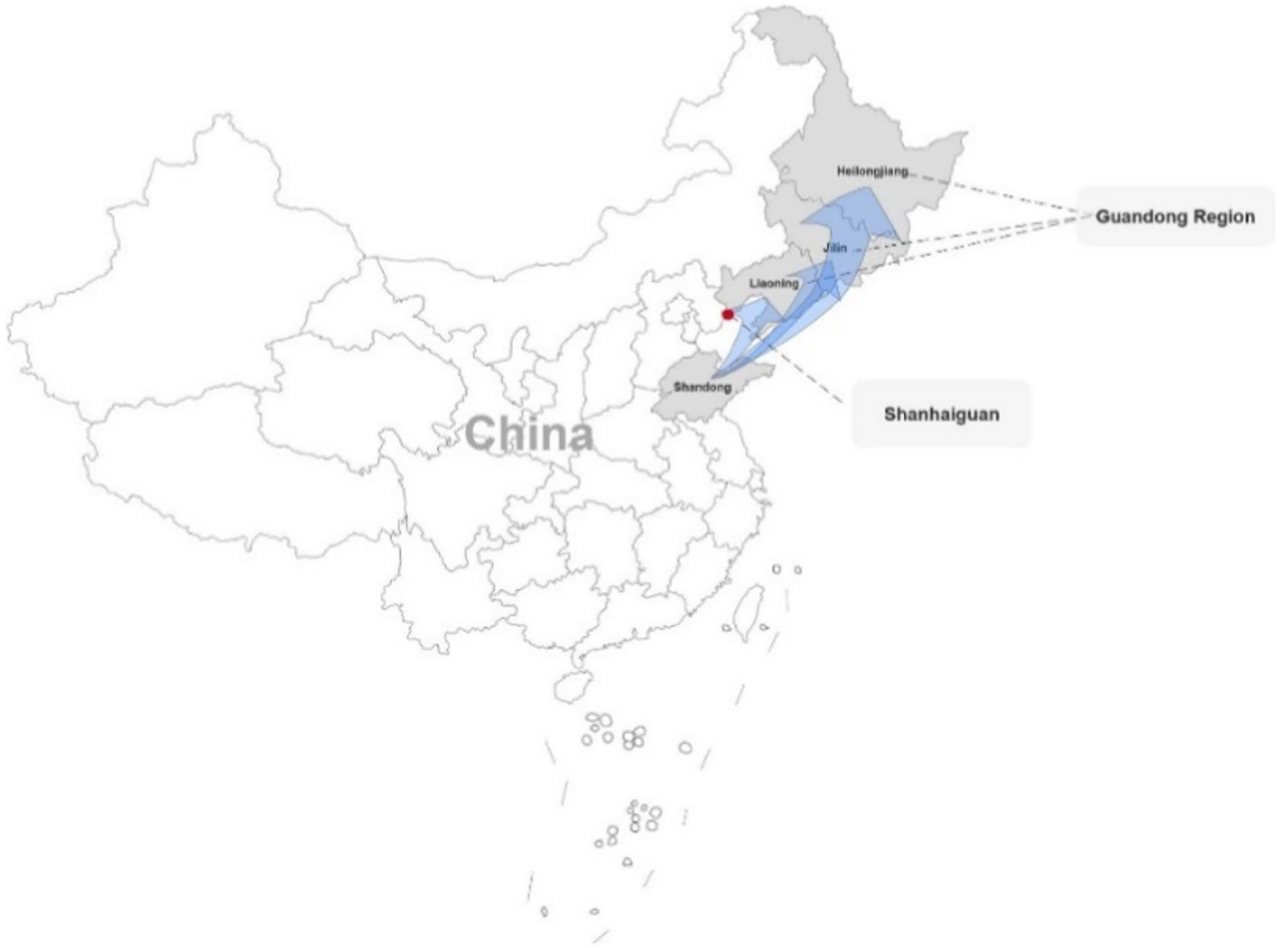
Map of Guandong region.

As a voluntary frontier settlement movement, it is possible that the Chuangguandong Movement could provide a better explanation for the formation of individualism in the northeast. Together with “Go West” and “Go South,” the Chuangguandong Movement is one of the three famous population migrations in modern China. It initiated the modern mass migration to Northeast China in 1860, which continued until the September 18 Incident in 1931. The “pass” of Chuangguandong refers to Shanhaiguan (Mountain-sea Pass; Fan, 2005), which marks the effective boundary between Northeast China and North China as shown in Figure 1. Northeast China is located in the east of the Shanhaiguan and includes Heilongjiang, Jilin and Liaoning Provinces, which is considered the “Guandong” region. The region of North China named Guannei mainly includes Shandong, Hebei and Henan Provinces. This is referred to as Shandong Province in this study, considering that 71% of Northeast China immigrants originated from Shandong in 1929 (Fan, 2005). Because Guannei residents actually illegaly inhabited Northeast China during the Qing Dynasty, which practiced the prohibition policy in the northeastern region called “Chuangguandong.” Even after the prohibition policy was lifted, the people still referred to the phenomenon of immigration from Guannei to the Guandong region as Chuangguandong (Zhang, 1998). The Chuangguandong Movement, which refers to the mass migration of people between 1860 and 1931 in this paper, was similar to the “Westbound Movement” in the United States. Before the Qing Dynasty, the population of Northeast China was especially modest, and most of the land in this region was barren and unexplored. In the first year of Tongzhi refers to 1862, there were only 3.16 million people (out of 255.41 million total people in China) in the northeast (Fan, 2005). By 1931, the total population of Northeast China reached 38.05 million (Gottschang and Lary, 2000), which was more than 12 times the population in 1860, and this increase was mostly caused by immigration, far exceeding the natural population growth rate. Although the figures recorded during this period varied for historical reasons, this was undoubtedly the largest migration flow in Chinese history (Zhang, 1998; Gottschang and Lary, 2000; Fan, 2005).

Taking the above historical context in consideration, it is necessary to establish whether the Chuangguandong Movement complied with the three distinct psychological processes of the voluntary frontier hypothesis. First, the settlements had the high motivation of immigrants. As is well known, North China was seen as overcrowded, impoverished and wretched. In particular, Shandong was terribly poor and brutally buffeted by nature. For 51 years (1860–1911), Shandong Province suffered natural disasters almost every year (Gottschang and Lary, 2000; Fan and Jia, 2016). In addition to natural disasters, frequent military disasters and bandits were other serious issue with which the people of North China had to contend. As the center of China’s political geography, North China became the base for various political forces. Conflicts such as the Zhi Wan War, Zhi Feng War, Central Plains War and others all brought huge losses to local farmers (Zhang, 1998; Fan, 2005). Moreover, there was a high population density in North China. E.g., in the 24th year of Guangxu refers to 1898, the population density of Shandong Province reached 246.24 people per square kilometer, which was six times the national average of 41.29 (Zhang, 1998; Fan, 2005). Nonetheless, the population of North China was still large and growing. From the Xinhai Revolution (1911) to the early 1930s, the population of Shandong increased from approximately 37 million to 40 million (Gottschang and Lary, 2000), but Shandong was constantly poor, apparently becoming even poorer as the population surged. Nevertheless, Guannei residents had to endure unimaginable tax exploitations. According to statistics, the best rice fields in China were only 0.4 per mu (1 acre is about 6 mu) in 1902, while Laiyang in Shandong Province increased nearly fivefold in 1927 (Zhang, 1998). When Zhang Zongchang took the post of military supervisor in Shandong, he financially exploited the people, leading to an extremely heavy tax burdens and skyrocketing bankruptcy rates. At that time, the people even expressed their anger through folk song lyrics: “Zhang Zongchang, come to Jinan, people have taxes, dogs have donations, one pot over 800 cost” (in Chinese, “张宗昌,来济南,人有税,狗有捐,一个锅头八百钱”) (Fan, 2005).

The heavy financial burden caused widespread hardship, and the peasants in North China eventually were forced to find and explore new places to live in order to survive. Northeast China met some of the wishes of immigrants, with a vast territory, abundant resources, and a sparse population. In 1928, the three northeastern provinces accounted for only 6.01% of the population, but 12.5% of the total land area (Fan, 2005). Until 1931, there was still a large amount of uncultivated land in Northeast China, with 5.67 square kilometers of arable land *per capita*, three times that of Shandong (Zhang, 1998). In addition, the soil was fertile, allowing for growth of rich crops such as soybeans, sorghum, barley, and corn as well as cash crops such as bananas, sugarcane, and litchi (Zhang, 1998; Gottschang and Lary, 2000). Accordingly, Japan considered Northeast China to be the treasure trove of East Asia, while Europe and the United States called it the “New Continent” of Asia (Gottschang and Lary, 2000). As discussed above, the abundant resources and free lands attracted settlers. Although, due to poverty and difficult conditions, some starved to death or died of disease during migration, this ultimately could not stop the will and determination of the immigrants.

Second, immigration to the Northeast increased sharply over time. From 1891 to 1911, the population in three Northeastern Provinces increased from more than 5 million to 19.19 million, with at least 10 million immigrants in Guannei (Fan, 2005). From the founding of the People’s Republic of China in 1912 to the “September 18 Incident” in 1931, the total population of people in the three northeastern provinces increased from 21,694,193 to 29,073,049. In just 20 years, the population increased by more than 7 million, which was far beyond the natural population growth rate (Zhang, 1998; Fan, 2005). Later, some researchers estimated that the actual number of immigrants may have been more than what the officially statistics reported (Ho, 1959; Gottschang and Lary, 2000). This might be true not only with respect to the numbers of migrants but also with respect to the total population size. According to the statistics, from 1891 to 1931, the average annual floating population of Shandong Peninsula was 13.07 per thousand (Gottschang and Lary, 2000). Meanwhile, the population density was low in Northeast China for several historical and geographic reasons, as the region was completely isolated from China due to its few natural transportation routes (Gottschang and Lary, 2000). According to Wu Xiyong’s statistics, in the early Republic of China (1912), the average population density was 14.1 people per square huali (1 huali is equal to 0.5 km) in Liaoning Province, 5.6 people per square huali in Jilin Province, and only 1.7 people per square huali in Heilongjiang Province (in the 24th year of Guangxu (1898), while the average population density was only 82.58 per square huali) (Fan, 2005).

However, after many Guannei immigrants arrived in the northeast, what awaited them was not a comfortable living environment but instead survival challenges of all types (Zhang, 1998; Gottschang and Lary, 2000; Fan, 2005; Fan and Jia, 2016). The natural conditions in Guandong were different from the ones in Guannei. For example, the northeast region had a continental monsoon climate, with long and cold winters, with temperatures below zero for more than 5 months. Therefore, since ancient times, the northeast was regarded as a bitter cold place on the frontier, scarcely populated. Moreover, the ecological environment was relatively primitive, and wild beasts frequently appeared. Even so, the residents overcame many difficulties and chose to stay in this region.

Third, Northeast China was inhabited by a huge number of voluntary settlers, which shaped a culturally shared lay theory of behavior. In Northeast China, various ethnic groups lived together, which promoted the mutual economic and cultural exchanges. The Han nationality became the main ethnic group except Manchuria, Mongolia, and Korea. This was fundamentally different from the culture formation of the Westward Movement in the United States, which directly replaced the previous culture by means of destruction, while the northeast was formed on the basis of equality and peaceful exchange and integration (Xu and Wang, 2016). It was not only in the material culture field and the cultural practice field but also in the spiritual field, Northeast China reflected the immigrant culture with regional characteristics (Fan and Jia, 2016; Fan, 2021), which constituted the three key factors of cultural spirit, namely explicit cultural values, cultural practices and implicit psychological tendencies (Kitayama et al., 2010). Although more than 100 years have passed, the northeast immigrant culture still reflects this state of multiethnic coexistence with the Han nationality as the theme. Northeast food, humor, art of paper-cut, song and dance duets, cheongsam, and the national spirit of self-improvement, hard work, self-reliance, and cooperation that can be observed in the northeast are still passed down from generation to generation (Kitayama et al., 2010; Fan and Jia, 2016; Fan, 2021; Ren et al., 2021).

Some scholars used independent/interdependent measures, such as self-construal scale, symbolic self-inflation, nepotism tasks, and percentage of most common names, to examine residents of Heilongjiang and Shandong, revealing that contrasted to Shandong Province, Heilongjiang residents had lower levels of interdependence and in-group preference, but the self-inflation was higher, and a greater preference for giving their children unique names. These results suggested that the differences between Heilongjiang and Shandong people in terms of independence may be related to the Chuangguandong Movement (Bai and Ren, 2021). However, this study was limited by the research method, while the included participants were relatively subjective in interpreting the meaning of the scale, which varied from person to person and was characterized by uncertainty. In terms of the representativeness of the regional population, due to the small sample size, the total number of subjects was only 179 (104 in Heilongjiang and 75 in Shandong), which may affect the stability of the research results. Considering the problems of strong subjectivity and small sample size in existing research, this issue should be further explored by using a more suitable data acquisition channel, which is also one of the contributions of this study.

As society has progressed, social media have become one of the most popular online activities and an integral part of daily internet usage. In their study, Na et al. (2015) investigated Facebook users from 49 countries and conducted a multicultural analysis, finding that users from individualist countries had more self-centered networks (i.e., network members were connected through the self) compared to users from collectivist countries. As these studies were proven to be valid and stable, we also decided to use Chinese social media tools to overcome the limitations associated with subjectivity and inadequate sample size of content measurement in previous studies.

Sina Weibo is the mainstream social media platform in China, which is based on the sharing, dissemination, and access of user relationship information through a “follow” mechanism and allows users to share information interactively and instantaneously through a variety of mediums such as text, pictures, videos, and other forms. Since its launch and promotion in August 2009, it underwent explosive growth, reaching 511 million active monthly users and 224 million active daily users as of September 2020 (Center Sina Weibo Data, 2021). As they are publicly available, Sina Weibo data are easy to obtain and can be used for scientific research in several fields. As of April 14, 2022, there were 5,987 papers on the topic “Sina Weibo” in China National Knowledge Infrastructure (CNKI). There are also psychological studies based on microblog big data. For example, based on the big data of 1 million active Weibo users, Ren et al. (2017) used keywords related to individualism/collectivism, compared provincial differences in individualism, and collectivism and confirmed the effectiveness of the big data approach. Therefore, using the text data of Sina Weibo from 2010 to 2020, combined with the dictionary of individualism/collectivism (Ren et al., 2017), we investigated whether individualism in Northeast China was higher than that in Shandong, using the framework of the voluntary frontier settlement hypothesis for testing.

## 2. Data and method

### 2.1. Data collection

**Figure 2:**
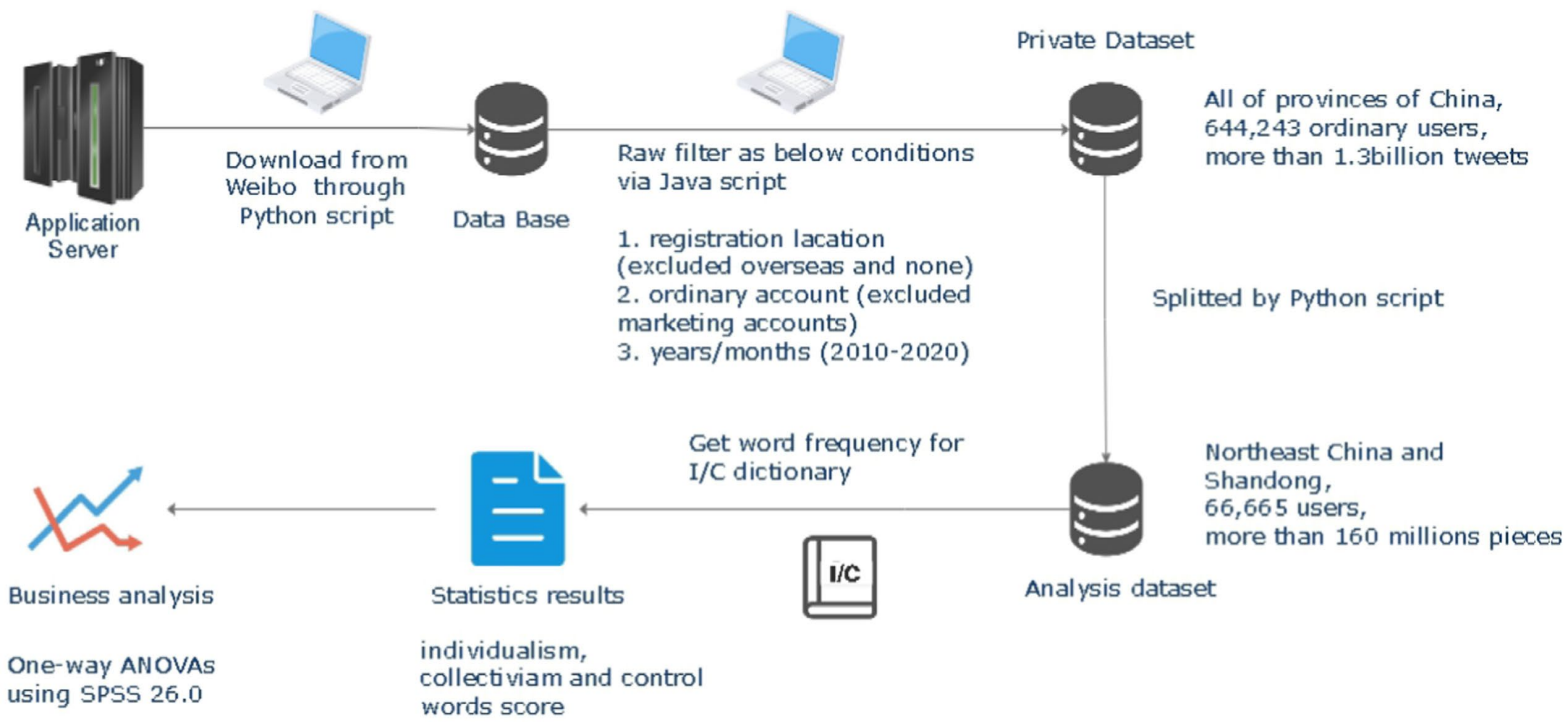
The procedure from data collection to statistical analysis.

The present study included more than 500 million active users of Sina Weibo. We downloaded 11 years of microblogging content from January 2010 to December 2021 through Weibo’s public application service interface API, covering 31 provinces and autonomous regions in China. Figure 2 depicts the process from data collection to statistical analysis in this study. The details on indicator selection, data cleaning and variable calculation are described in the corresponding section. The research methods and used procedures were approved by the Research Ethics Committee of the Institute of Psychology, Chinese Academy of Sciences, with ethical specification H15009.

### 2.2. Word selection

This study was based on original user-posted content on Sina Weibo, which was used to analyze the frequency of individualism/collectivism keywords that were determined based on relevant social and cultural psychology studies. The construction of the individualism/collectivism dictionary was based on the Chinese vocabulary that was identified following group discussion of domestic scholars, and it included 53 individualist words such as “you,” “I,” “he/she” and 112 collectivist words such as “we,” “you,” “family” etc. the first-person called singular and plural pronouns (Hamamura and Xu, 2015; Zeng and Greenfield, 2015), economic priority and personal property words. At the same time, some neutral words, such as “of,” “and” and other auxiliary words, conjunctions and so on, were also sorted out and refined as baseline words to calibrate the deviation of individualism/collectivism keyword statistics. This method has been effectively verified by comparing the regional differences in individualism/collectivism at the provincial level (Gao et al., 2013), and found that the outbreak of COVID-19 increased collectivism and reduced individualism through Weibo data based on the parasite theory of collectivism (Ren et al., 2020).

### 2.3. Statistical analysis

We first obtained the original microblogging text data through the JAVA program and distinguished the province and prefecture where the subjects were located by the user’s registration place. Although there may be some information inconsistency between the registration place and the real place of residence, which is difficult to verify, the distorted data become diluted when the amount of data is large enough. To ensure the authenticity of the data as much as possible, we eliminated the accounts that had no place of registration and were identified as overseas. Since Weibo does not have fields for users’ birthplace and permanent residence, it is difficult to distinguish between natives and outsiders. However, this did not affect the analysis of our results (Fan, 2005; Luo and Ren, 2018). Second, in the selection of users, marketing and authentication accounts were excluded and more than 1.3 billion tweets of Weibo data from 644,243 ordinary users who published original Weibo content (263,990 were male, accounting for 41%) were included. There were many missing values in the age information, accounting for 78.89%. Among the remaining users with age information, most were aged 28–38, accounting for 13.23%, followed by users aged 18–28, accounting for 5.7%, and 38–48 years old and 48–58 years old, accounting for 1.78 and 0.31%, respectively. There were very few users ≥ 58 years old, accounting for 0.1%. Since this study analyzed users in Northeast China and Shandong, 160 million pieces of microblog content with 66,665 users registered in Heilongjiang (15,872), Jilin (9,803), Liaoning (17,988) and Shandong (23,002) were extracted from the microblog user pool. Next, individualism/collectivism was calculated as a within-groups variable and treated province was treated as a between-groups variable when computing descriptive statistics and conducting one-way ANOVAs. To control the impact of changes in the total number of microblog users and balance the large differences in the number of microblog users and posts in various provinces, the frequency of each word was calculated according to the social cultural and psychological dictionary. Then, the ratio of the frequency of keyword words to the number of microblogs published in that month was used as the score of individualism, collectivism, and control words in the three northeastern provinces and Shandong. After that, the monthly individualism, the monthly collectivism, and the monthly control word score obtained were summed up and averaged, and the annual average individualism, collectivism, and control word score for the 11 years from 2010 to 2020 were finally obtained. To minimize the bias between individualism and collectivism in the four provinces, we divided the annual average individualism and collectivism score by the annual average control word score, respectively. Next, the difference between the two was analyzed as an individualism index. A positive value represents strong individualism and vice versa. SPSS 26.0 was used for statistical analyzes.

## 3. Results

**Figure 3:**
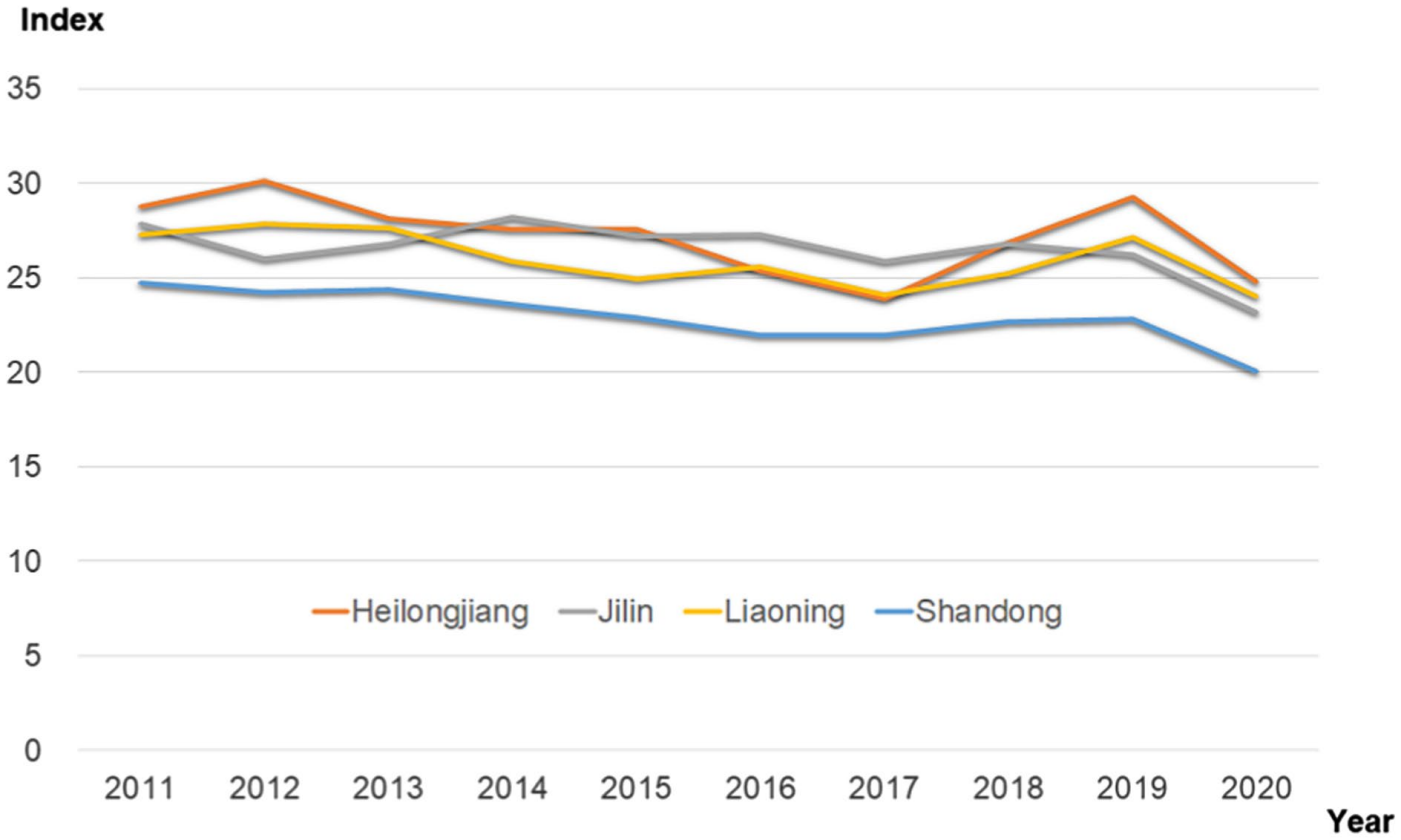
Trend of individualism index. It is the visual presentation of Table 1, data source from Weibo’s public application service interface API, the Weibo posts used in the present study are publicly available, while users’ privacy is strictly protected according to ethical principles. Individualism index refers to the subtraction of collectivism from individualism.

Through a series of preprocessing steps on the original individualism and collectivism score, we finally obtained the individualism index. Since the calculated scores were too small, we uniformly multiplied the scores by a factor of 100 (i.e., individualism index*100) to check for cultural differences in individualism between the two locations as shown in Table 1. The annual changes in differences in the individualism index between the Guandong region and Shandong are shown in Figure 3.

A one-way ANOVA with province as the between subjects variable revealed a significant main effect as shown in Table 2, *F*(3,40) = 15.85, *p* < 0.01, *η*^2^ = 0.54. Post-hoc comparisons using the Tukey HSD test revealed that Heilongjiang (*M* = 27.12, SD = 1.97) was more individualistic than Shandong (*M* = 22.90, SD = 1.33), *t*(20) = 5.89, *p* < 0.01, Cohen’s d = 2.51. This was also the case when comparing Jilin (*M* = 26.43, SD = 1.38) to Shandong (*M* = 22.90, SD = 1.33), *t*(20) = 6.10, *p* < 0.01, Cohen’s d = 2.60, and when comparing Liaoning (*M* = 25.84, SD = 1.42) to Shandong (*M* = 22.90, SD = 1.33), *t*(20) = 5.01, *p* < 0.01, Cohen’s d = 2.14. In summary, the three provinces of Heilongjiang, Jilin and Liaoning had a higher tendency toward individualism compared to the reference Shandong province, particularly Heilongjiang province, which was found to have the highest tendency in Northeast China. These results showed that even after expanding the sample size and establishing the true meaning of the expression, the same results were obtained, which is consistent with Bai and Ren (2021) findings. These results validated our research hypothesis based on the framework of voluntary frontier settlement, illustrating that this phenomenon may be related to the influence of the Chuangguandong Movement.

**Table 1:** The individualism index* by provinces.

Year	Individualism index*			
	Heilongjiang	Jilin	Liaoning	Shandong
2010	25.92	25.36	24.61	22.67
2011	28.81	27.82	27.27	24.73
2012	30.16	26.01	27.84	24.23
2013	28.14	26.80	27.66	24.38
2014	27.57	28.21	25.87	23.58
2015	27.57	27.20	24.93	22.88
2016	25.35	27.28	25.59	21.94
2017	23.87	25.84	24.10	21.95
2018	26.85	26.79	25.22	22.68
2019	29.25	26.21	27.13	22.80
2020	24.80	23.17	24.03	20.06

*Individualism index refers to the subtraction of collectivism from individualism.

**Table 2:** The result of one way ANOVA for Individualism index.

C	Sum of squares	df	Mean square	*F*	Sig.
Between groups	113.63	3	37.88	15.85	0.00
Within groups	95.58	40	2.39		
Total	209.21	43			

*F*(3,40) = 15.85, *p* < 0.01.

## 4. Discussion

Based on the Weibo text data that were collected for period from 2010 to 2020, this study used an individualistic/collectivist dictionary to extract text features and analyze the word frequency. One-factor variance verification revealed that the individualism tendency of Heilongjiang, Jilin, and Liaoning in the Chuangguandong region was higher than that in Shandong Province. By excluding other ecological factors, the Chuangguandong Movement was confirmed as a possible cause of the highly individualistic culture of the northeast region. This is consistent with the research conclusion of Bai and Ren (2021), which further confirmed the robustness of the findings and the universality of the explanation of the influence of the voluntary frontier movement on individualism and verified the effectiveness of the method. These results also provide new evidence for the future study of Chinese individualism and cross-cultural research. Moreover, we also found that regardless of the year, the individualism in the three northeastern provinces was higher than that of Shandong, which suggested that individualistic tendencies in the northeast region were very stable.

### 4.1. Ecology of individualism

There are many factors influencing the regional differences in individualism/collectivism that could further explain the possible impact of voluntary frontier movement in Northeast China. Other ecological factors are shown and compared in Table 3. (1) Modernization theory: the *per capita* GDP in Northeast China (42,635 in Heilongjiang, 50,800 in Jilin, and 58,872 in Liaoning) was less than that of Shandong Province (72,151) (Department of Heilongjiang statistical, 2021; Department of Jilin Statistical, 2021; Department of Liaoning Statistical, 2021; Department of Shandong Statistical, 2021). According to modernization theory, the level of individualism in Northeast China was lower than that in Shandong, which had a higher degree of economic modernization. This is obviously contrary to our analytical conclusions. Therefore, modernization theory cannot explain the phenomenon of a highly individualistic culture in Northeast China well. (2) Climate economic theory: the climate demand of the three northeastern provinces (Heilongjiang was 89.29, Jilin was 82.71, and Liaoning was 70.75) was higher than that of Shandong (51.63) (NOAA, 2022), and in terms of economic indicators, the *per capita* GDP of the three northeastern provinces was lower than that of Shandong. Taking in consideration this theory, it was found that the climate environment in the northeast was relatively poor and the economic resources were relatively weak, so the collectivist tendency in the northeast was stronger than that in Shandong. Again, this was contrary to our conclusions. Therefore, this theory cannot be considered as possible explanation for the higher levels of individualism observed in Northeast China than in Shandong. (3) Rice theory: the percentage of rice planted in Northeast China (26.82% in Heilongjiang, 14.73% in Jilin, 14.75% in Liaoning) was larger than that in Shandong (1.36%) (Department of Heilongjiang statistical, 2021; Department of Jilin Statistical, 2021; Department of Liaoning Statistical, 2021; Department of Shandong Statistical, 2021). According to this theory, individualism in the northeast was lower than in Shandong. Therefore, the rice theory also does not provide a reasonable explanation for the findings of the present study.

**Table 3:** Ecology of individualism.

Index	Heilongjiang	Jilin	Liaoning	Shandong
Climatic demands	89.29	82.71	70.75	51.63
*Per capita* GDP 2020	42,635	50,800	58,872	72,151
Rice	26.82%	14.73%	14.75%	1.36%

Climatic demands is the sum of the absolute deviations from 22°C (ca. 72°C) for the lowest and highest temperatures in the coldest month and the lowest and highest temperatures (Van de Vliert, 2011). Data source from Meteorological data from 1929–2021 in NOAA. Per capita GDP and Rice, which provide data from statistical yearbooks of each province (Department of Heilongjiang statistical, 2021; Department of Jilin Statistical, 2021; Department of Liaoning Statistical, 2021; Department of Shandong Statistical, 2021).

Comparing the above with possible competitive ecological factors, we found no support for the above factors, and they could not explain the phenomena observed in this study. However, the voluntary frontier settlement hypothesis proposes that regions with frontier settlement are more individualistic (Turner, 1920). As mentioned earlier, the theoretical framework includes three important processes, i.e., self-selection, reinforcement, and institutionalization (Kitayama et al., 2006; Bai and Ren, 2021), which not only explain the individualism presented in the United States compared to other Western European and East Asian countries (Kitayama et al., 2009), but also the differences between Hokkaido and the main island of Japan (Kitayama et al., 2006) and the differences between Shenzhen and other reference regions (Chen et al., 2016, 2019; Luo and Ren, 2018). As a voluntary frontier settlement in China, the Chuangguandong Movement includes the three processes mentioned above, i.e., the high immigrants’ motivation (Zhang, 1998; Gottschang and Lary, 2000; Fan, 2005; Fan and Jia, 2016), the continuous influx of immigrants’ adaptation to the ecological conditions in Northeast China (Ho, 1959; Zhang, 1998; Gottschang and Lary, 2000; Fan, 2005), and the gradual formation of an independent spirit in the northeast region (Fan, 2005, 2021; Fan and Jia, 2016), all of which reflect the independent tendency brought about by the Chuangguandong Movement. Therefore, the Chuangguandong Movement provides better clarification of these historical events, which may explain why the northeast region is more individualist than Shandong. However, more research is needed to further verify the reported findings.

The ideal way to manipulate the independent variables through experimental design would be to test the changes in the individualism of Northeast China before and after the Chuanguandong Movement. Nevertheless, the Chuangguandong Movement is a historical event, so it is impossible to make before and after comparisons. Moreover, it is also difficult to manipulate other social and ecological factors in the laboratory. Therefore, most of the current studies used historical events as antecedent variables to examine the differences in individualism between the two places through comparative analysis of the historical event and the reference place.

### 4.2. Online social media and dictionary

By using the method of extracting word frequencies based on microblog big data and the individualism/collectivism dictionary, we reached conclusions that are consistent with the research results reported by Bai and Ren (2021). This proved the effectiveness of this method and provided a new research channel for the study of individualism/collectivism. On the one hand, Weibo, as one of the popular social platforms in China, reflected the representativeness of the sample, the objectivity of the content, the convenience and timeliness of data acquisition and so on. The big data from Weibo include a much larger sample size and more provinces (160 million text data for Heilongjiang 15,872, Jilin 9,803, Liaoning 17,988 and Shandong 23,002, respectively) compared to the sample size of only 179, Heilongjiang 104, and Shandong 75 used in study by Bai and Ren (2021), which to some extent reduces the error caused by sample size. Since our data included decade-long data from 2010 to 2020, this approach has more advantageous in terms of stability in reflecting regional differences and ability to further improve the robustness of the analysis results. With the development of social media, people are increasingly more inclined to use pictures and video materials to express their emotions and psychological states, so future studies should also consider feature extraction and analysis based on pictures and video materials. On the other hand, the iterative individualism/collectivism dictionary was effectively verified again. In this study, we did not consider the relevant extended words in the dictionary when processing text features. E.g., in the individualist vocabulary, we only considered “I” (in Chinese, “我”) but did not include its extended word “Wu” (in Chinese, “吾”). In our future work, we plan to use Chinese text analysis software named the “Wenxin” system (Zhao et al., 2016) when extracting text features, as this approach has already been verified, and it might produce different findings.

### 4.3. Limitations

Although this study solved the problems of small sample size and subjective content encountered in previous studies, there are still some areas for improvement and refinement. First, in terms of sample selection, since most of the microblog users were 28–38 years old and the sample of users in other age groups was relatively small, it remains unclear whether these data truly reflect the people in the Guandong region, which should be comprehensively analyzed in the future by combining data from other channels. However, such problems were also encountered in previous studies. When Chen et al. (2016) analyzed independent self in Shenzhen, the subjects were 238 high school students from Shenzhen and Foshan. The second issue is in the control of individual-level factors, since gender and age are not mandatory fields for Weibo users, the missing values accounted for a large proportion, making it impossible to effectively control these influencing factors in the analysis. This issue can be solved by technical means in future data extraction and preprocessing. Third, the triangulation model mentioned in Kitayama et al. (2006) could be considered to raise the effectiveness of the research method. Since the data we obtained came from Weibo, where the main users were Chinese, and users’ locations in other counties were marked as overseas, it was difficult to distinguish a specific country. Thus, it would be useful to obtain the data from other social platforms such as Twitter, to verify the consistency of the two platforms. However, it does not affect the effectiveness of our research method. In Japan’s independent culture studies, Ishii (2014), Ishii et al. (2014) considered the different consequences of voluntary settlement were closely related to the specific culture in which voluntary settlement occurred. This means that, since the different cultural backgrounds between Northeast China and the United States, even if the United States is taken into account as the control group, it still cannot not change the fact that the results of the individualism in the Northeast China are higher than those in Shandong. Fourth, the trend of individualism in the past 11 years did not have a horizontal trend, but it fluctuated with years. Future studies should further analyze the reasons or influencing factors of these fluctuations. Fifth, as is well known, after the economic system reform in the 1980s, Northeast China changed from the original net population inflow to outflow region. Especially after the middle 1990s, the speed of the net migration of the population accelerated. Therefore, it is also worth further discussing whether the microblog data after 2010 should be analyzed in combination with population mobility.

## 5. Conclusion

Our results showed that inhabitants in Northeast China, an area affected by the Chuangguandong historical movement, were more individualistic than their counterparts in Shandong. This finding supported that the Chuangguandong Movement which could be constructed as a historical voluntary frontier settlement in China fostered independent agency, thus, extending and enriching the generalization of the frontier ecology of individualism.

## Data availability statement

The raw data supporting the conclusions of this article will be made available by the authors, without undue reservation.

## Ethics statement

The studies involving human participants were reviewed and approved by the Research Ethics Committee of the Institute of Psychology, Chinese Academy of Sciences. The patients/participants provided their written informed consent to participate in this study.

## Author contributions

XR designed the study and provided critical revision and full guidance of the paper. YW collected and analyzed the data and wrote the manuscript with input from all authors. All authors contributed to the article and approved the submitted version.

## Funding

This research was funded by the National Social Science Fund of China project (Grant No. 20BSH142) and the Strategic Priority Research Program of Chinese Academy of Sciences (Grant No. XDA27000000).

## Conflict of interest

The authors declare that the research was conducted in the absence of any commercial or financial relationships that could be construed as a potential conflict of interest.

## Publisher’s note

All claims expressed in this article are solely those of the authors and do not necessarily represent those of their affiliated organizations, or those of the publisher, the editors and the reviewers. Any product that may be evaluated in this article, or claim that may be made by its manufacturer, is not guaranteed or endorsed by the publisher.
